# Cellulose-Derived Battery Separators: A Minireview on Advances Towards Environmental Sustainability

**DOI:** 10.3390/polym17040456

**Published:** 2025-02-09

**Authors:** Tayse Circe Turossi, Heitor Luiz Ornaghi Júnior, Francisco Maciel Monticeli, Otávio Titton Dias, Ademir José Zattera

**Affiliations:** 1 Post-Graduate in Process Engineering and Technologies Program, University of Caxias do Sul, Francisco Getúlio Vargas St., Caxias do Sul 1130, RS, Brazil; tcturossi1@ucs.br (T.C.T.); ornaghijr.heitor@gmail.com (H.L.O.J.); ajzatter@ucs.br (A.J.Z.); 2 Department of Aerospace Structures and Materials, Faculty of Aerospace Engineering, Delft University of Technology, 2629 HS Delft, The Netherlands; 3 Centre for Biocomposites and Biomaterials Processing, Faculty of Forestry, University of Toronto, WillCocks St., 33, Toronto, ON M5S 3B3, Canada; otavio.dias@mail.utoronto.ca

**Keywords:** cellulose, battery separators, sustainability, technological innovation

## Abstract

Cellulose-derived battery separators have emerged as a viable sustainable alternative to conventional synthetic materials like polypropylene and polyethylene. Sourced from renewable and biodegradable materials, cellulose derivatives—such as nanofibers, nanocrystals, cellulose acetate, bacterial cellulose, and regenerated cellulose—exhibit a reduced environmental footprint while enhancing battery safety and performance. One of the key advantages of cellulose is its ability to act as a hybrid separator, using its unique properties to improve the performance and durability of battery systems. These separators can consist of cellulose particles combined with supporting polymers, or even a pure cellulose membrane enhanced by the incorporation of additives. Nevertheless, the manufacturing of cellulose separators encounters obstacles due to the constraints of existing production techniques, including electrospinning, vacuum filtration, and phase inversion. Although these methods are effective, they pose challenges for large-scale industrial application. This review examines the characteristics of cellulose and its derivatives, alongside various processing techniques for fabricating separators and assessing their efficacy in battery applications. Additionally, it will consider the environmental implications and the primary challenges and opportunities associated with the use of cellulose separators in energy storage systems. Ultimately, the review underscores the significance of cellulose-based battery separators as a promising approach that aligns with the increasing demand for sustainable technologies in the energy storage domain.

## 1. Introduction

In recent years, the number of electrochemical energy storage devices, such as batteries and supercapacitors [1], have been widely increased mainly due to their high energy density, power, and long lifespan. These devices have various applications, including in electric vehicles, computers, drones, and portable electronic devices [2]. Consequently, the quest for enhancing the electrochemical performance of these devices has generated significant interest in researching new materials and innovative structural designs [2,3]. There is also a growing interest in developing more sustainable and environmentally friendly components for these devices, such as current collector electrodes, electrolytes, and battery separators [2,4].

The separator serves as a physical barrier that effectively divides the positive electrode (cathode) from the negative electrode (anode), facilitating ion movement while simultaneously preventing short circuits [5]. This component, often termed a membrane or porous film, is designed to possess substantial mechanical strength to endure manufacturing stresses, an optimal thickness, thermal stability, and superior adsorption and retention of the electrolyte, along with high ionic conductivity [5]. Furthermore, it is essential for separators to demonstrate chemical resistance to endure interactions with the electrolyte contained within the battery [5,6].

The optimal performance of the separator is influenced by various properties, including structural, physicochemical, and functional aspects, as well as cost and sustainability considerations [3].

Table 1 summarizes the main properties of a battery separator.

Commercial batteries predominantly utilize separators composed of polyolefins, including polyethylene (PE) and polypropylene (PP). These polymers are favored for their considerable mechanical strength, chemical resilience, and cost-effectiveness. Nevertheless, their thermal stability is relatively low, attributed to the melting points of PE at 130 °C and PP at 165 °C. Additionally, the hydrophobic nature of these materials restricts the wettability of the membrane, which in turn diminishes ionic conductivity and overall performance [3].

The increasing scarcity of energy resources, coupled with pressing environmental concerns like carbon dioxide emissions and water contamination, has rendered the pursuit of sustainable and biodegradable alternatives to petroleum-based materials essential [1,2,3]. In response to these challenges, numerous strategies have been investigated to enhance the characteristics of separators. One notable strategy is the creation of hybrid separators that integrate additives to improve their functionality. These hybrid separators may provide considerable benefits compared to conventional commercial separators [3]. Additionally, the employment of green polymers as polymeric matrices represents another promising approach [7].

**Figure 1:**
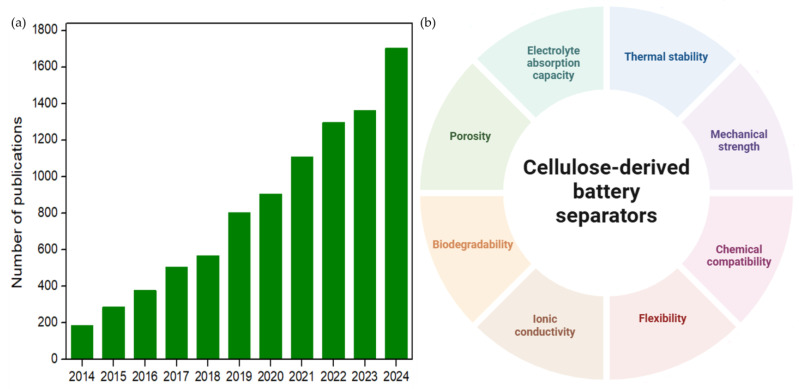
(**a**) The numbers of publications on cellulose separators in the last decade obtained from the ScienceDirect database and (**b**) main characteristics of cellulose-derived separators.

Cellulose has emerged as a viable alternative for high-performance and high-safety battery separators, attributed to its remarkable wettability, thermal dimensional stability, and non-toxic, environmentally sustainable properties. The utilization of cellulose at the nanoscale, particularly in the form of nanocellulose, can further enhance these beneficial characteristics [7]. Notably, the volume of scholarly publications concerning cellulose-based separators has surged by 819.5% from 2014 to 2024, indicating a significant increase in interest and progress within this research domain, as demonstrated in Figure 1 [8]. Additionally, the primary attributes of cellulose-derived separators, including porosity, thermal stability, mechanical strength, electrolyte absorption capacity, chemical compatibility, biodegradability, flexibility, and ionic conductivity, are illustrated, showcasing advancements in electrochemical performance.

The electrochemical enhancements offered by cellulose-based separators are intrinsically linked to their chemical composition. The presence of hydroxyl groups (-OH) throughout the cellulose structure imparts a natural hydrophilicity, which facilitates a strong attraction to water [9]. This property encourages the development of alternative routes for ion transport, thereby optimizing the retention and distribution of the electrolyte [9]. Furthermore, the potential for chemical functionalization of cellulose allows for a variety of chemical interactions with particular electrolytes, which further boosts ionic mobility [3,10,11,12]. The hydroxyl groups also contribute to improved wettability and a high electrolyte absorption capacity, ensuring enhanced ionic conductivity [9]. The inherent chemical versatility of cellulose enhances its compatibility with a range of electrolytes and electrode materials. Modifications to the surface chemistry can improve its resistance to degradation in electrochemical settings, while simultaneously fostering stable interfacial interactions, thereby prolonging the operational lifespan of energy storage systems [12,13].

Cellulose exhibits a three-dimensional morphology characterized by interconnected spaces, which leads to the development of porous structures [13]. The porosity of cellulose can be improved during the fabrication of separators by employing chemical functionalization and integrating various additives, facilitating the creation of pores at both micro- and nanoscale dimensions. This enhanced porosity plays a crucial role in optimizing ion transport and electrolyte uptake, thereby significantly influencing the performance of batteries [13,14]. Furthermore, the fibrillar network of cellulose provides high mechanical strength [14], making the separators robust and capable of withstanding operational stress, a crucial attribute for flexible battery applications [15].

Regarding thermal stability, cellulose-derived separators outperform synthetic polymers due to the natural thermal resistance of cellulose [14]. Cellulose maintains its structural integrity even at elevated temperatures, minimizing the risk of thermal shrinkage or degradation, thereby significantly enhancing battery safety [16,17]. One of the most notable benefits of cellulose separators is their biodegradability. In contrast to synthetic polymers, cellulose undergoes natural decomposition in environmental settings, thereby responding to the pressing necessity of mitigating the environmental consequences associated with battery disposal [12,18]. This combination of electrochemical, mechanical, thermal, and environmental properties positions cellulose as a promising and sustainable solution to the challenges faced by next-generation energy storage technologies.

Cellulose presents several advantages over traditional separators in terms of various properties. According to the findings of Wang, Lu, and Zhang [9], the presence of hydroxyl groups in cellulose facilitates enhanced ionic mobility by improving the retention, distribution, and wettability of electrolytes. Additionally, its inherent chemical versatility enables functionalization, which contributes to increased compatibility and stability within electrochemical settings. Zhang, Wang, and Liang [15] emphasized that the fibrillar network of cellulose forms a porous structure, optimizing ion transport, electrolyte absorption, and mechanical strength. Han et al. [17] highlighted its superior thermal stability compared to synthetic polymers, minimizing risks of shrinkage and degradation at high temperatures. Additionally, Zhang et al. [18] reported that its biodegradability provides an environmental benefit, addressing the need for sustainable energy storage solutions. These combined properties establish cellulose as a high-performance, eco-friendly material for next-generation batteries.

This article provides a comprehensive review of recent advances in cellulose-derived separators and hybrid separators with nanocellulose for battery applications, highlighting their role in advancing environmental sustainability. The discussion begins with the fundamental properties of cellulose and its derivatives, laying the foundation for understanding their role in energy storage. This is followed by an in-depth examination of key studies to elucidate the use of cellulose-derived separators, particularly in terms of their energy storage properties and performance. The discussion then moves to the critical issue of environmental impact and sustainability, where the life cycle of cellulose-based separators and their carbon footprint are assessed through recent studies. These findings highlight the potential of cellulose as a sustainable alternative. Finally, the article looks at the challenges and future prospects for integrating cellulose-based technologies into next-generation energy storage systems.

## 2. Cellulose and Its Derivatives

**Figure 2:**
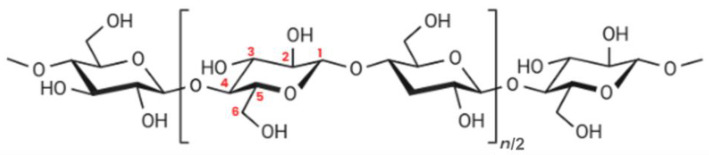
Chemical structure of cellulose. Red numbers represent the position of the carbons within the cellulose chain.

Cellulose, found in plants, bacteria, tunicates, and algae, is the most abundant biopolymer in nature, constituting approximately 40 to 50% of terrestrial biomass [7,17]. It consists of long linear chains of β-D-glucose units linked by β-1,4-glycosidic bonds, forming a repeating unit known as cellobiose (Figure 2). The general formula of cellulose is (C_6_H_10_O_5_)_n/2_, where ‘n/2’ represents the degree of polymerization, influenced by the source material [7,10,11,12].

This natural polymer can be processed by physical, mechanical or chemical methods to produce a variety of derivatives, including cellulose nanofibrils (CNFs), cellulose nanocrystals (CNCs), and bacterial cellulose (BC), each with unique properties and applications [7]. In addition, cellulose can be chemically treated to produce regenerated cellulose (RC) or derivatives such as cellulose acetate (CA) [11,19,20].

The following paragraphs will detail each type of cellulose separately and their prospects for use in battery separators. This section aims to summarize the key properties of each type of cellulose and highlight the improvements they enable in terms of performance and functionality. This summarized overview serves as a precursor to the detailed discussion in Section 3, where the specific enhancements associated with each type of cellulose will be contextualized based on relevant studies.

### 2.1. Cellulose Nanofibrils

CNFs are produced from the mechanical defibrillation of natural cellulose, resulting in fine long fibers with diameters less than 100 nm. Due to their low production cost and good mechanical properties, they are commonly used as reinforcement in composites [11,19].

In the context of battery separators, cellulose nanofibers (CNFs) serve as additives in hybrid polyethylene or polypropylene matrices, thereby improving the mechanical strength, flexibility, thermal stability, and ionic conductivity of these separators relative to conventional options [11]. Additionally, the incorporation of CNFs enhances the retention of electrolytes, which can lead to superior electrochemical performance in batteries [11,21].

### 2.2. Cellulose Nanocrystals

CNCs are produced by acid hydrolysis using H_2_SO_4_ and HCl, which separates the crystalline regions from the amorphous regions of cellulose [7]. The resulting crystals appear as crystalline solids with diameters ranging from 10 to 30 nm and lengths from 5 to 200 nm [11]. In addition to the morphological differences, CNC production is more time-consuming and costly than CNF production [19]. In addition to acid hydrolysis, oxidizing agents such as the TEMPO reagent (N-oxyl-2,2,6,6-tetramethylpiperidine) can be used to functionalize CNC surfaces by introducing carboxyl groups, improving their stability in aqueous dispersions and increasing their functionality [11].

Like cellulose nanofibrils, CNCs are widely used as additives in hybrid separators due to their high crystallinity and ability to reinforce the separator matrix [11]. Additionally, the high crystallinity of CNCs can provide better control over the separator’s porosity, thereby increasing ionic conductivity [11,22].

### 2.3. Bacterial Cellulose

Bacterial cellulose (BC) differs from conventional cellulose forms, such as CNFs and CNCs, as it is produced by bacteria like Gluconacetobacter xylinus, which release protofibrils from their cell walls [13]. These protofibrils subsequently aggregate into bundles, resulting in the formation of nanofibrils that create a highly porous and interconnected network structure characteristic of BC. This network is structured into a thin membrane of nanofibrils that develops on the surface of the culture medium [13]. These cellulose fibers exhibit excellent crystalline organization, with diameters ranging from 20 to 100 nm [23]. Chemically, bacterial cellulose is similar to plant cellulose but has higher crystallinity (95%) compared to CNFs and CNCs, which contain only about 65%, as well as a unique network of nanofibers in a three-dimensional mesh [13,24].

The main characteristics of BC are its large surface area, high permeability, elasticity, flexibility, tensile strength, and durability [23]. These properties make BC a promising renewable option for use as battery separators, as it can withstand high temperatures and prolonged charge/discharge cycles [23]. Studies indicate that bacterial cellulose incorporated with conductive additives can enhance energy efficiency and prolong the lifespan of batteries [24,25,26].

### 2.4. Cellulose Acetate

Cellulose acetate is obtained through the esterification of cellulose with acetic acid and acetic anhydride, using sulfuric acid as a catalyst [27]. This process classifies CA as a derivative of cellulose, as it alters its chemical and physical properties, transforming it into a thermoplastic polymer with applications in films and membranes [27].

CA can be used in the production of battery separators, as it can form thin, porous films with good thermal stability [28]. Additionally, it exhibits good electrical insulation, making it ideal for separators, especially in lithium-ion batteries. Furthermore, it can be modified to improve its electrochemical properties. Various studies report the use of CA as battery separators [28,29].

### 2.5. Regenerated Cellulose

Regenerated cellulose is produced via chemical methods that entail the coagulation of natural cellulose in a non-solvent medium, subsequently leading to its regeneration into various forms, including fibers, films, membranes, spheres, hydrogels, aerogels, and bioplastics, among others. An additional advantage of this technique is that the majority of solvents and coagulants employed in the synthesis of regenerated cellulose are recyclable, thereby rendering the process environmentally sustainable [9].

Due to its good flexibility and ability to form thin porous membranes, this type of cellulose is a viable alternative for use as battery separators [9]. Regenerated cellulose separators are lightweight and biodegradable and can enhance the performance and lifespan of batteries, particularly in portable devices [30].

The main advantages and disadvantages of cellulose derivatives as battery separators are summarized in Table 2.

As shown in Table 2, the specific type of cellulose derivative has an impact on the performance of battery separators. For example, the increased mechanical strength and thermal stability of CNFs contribute to the durability and safety of separators, ensuring structural integrity under operational stress [19]. Similarly, the controlled porosity and improved ionic conductivity of CNCs enhance ion transport, resulting in improved battery efficiency and reduced internal resistance [11]. BC’s exceptional thermal stability and potential for integration of conductive additives enables a long cycle life and energy efficiency improvements critical for high-performance applications [23]. This has led to bacterial cellulose being extensively studied in the scientific literature; however, its industrial use remains limited due to the slow production process and high cost, which remain significant drawbacks. The flexibility and electrical insulation of CA make it a suitable candidate for separators in systems requiring adaptability and electrochemical stability [28]. Finally, the porosity and flexibility of RC improves ion diffusion and separator lifetime, making it a promising choice for sustainable and high-capacity energy storage devices [9]. The different performance results of these cellulose derivatives are strongly influenced by their structural characteristics and the preparation processes used. The correlation between these factors determines the effectiveness of each material in applications such as battery separators, highlighting the importance of selecting the most appropriate material based on the specific needs of the batteries. In the next section, the performance of different types of cellulose derivatives will be discussed through a detailed review of scientific literature. This includes an analysis of their structural, thermal, and electrochemical behavior.

## 3. Performance of Cellulose-Derived Battery Separators

Driven by the quest for a more efficient use of sustainable natural polymers, cellulose has emerged as a promising alternative material compared to petroleum-based polymers, due to its abundance, sustainability, biodegradability, and biocompatibility [12,20]. Recent studies underscore an increasing focus on cellulose-derived separators for lithium-ion batteries, which is largely due to their superior wettability and thermal stability. As mentioned earlier, cellulose is available in multiple derivatives and forms, resulting in extensive research and investigation into its use in battery separator applications [29].

The manufacturing methods for cellulose-based separators vary, encompassing techniques such as coating commercial separators, casting, electrospinning, vacuum filtration, and phase inversion [3]. Each method imparts unique characteristics to the separators. For example, casting and coating offer ease of processing and the potential for continuous production, but may result in low thickness uniformity [29,31]. In contrast, phase inversion allows for the production of uniformly porous separators on a large scale, although the process parameters are more complex. Electrospinning generates separators with high porosity and good structure, albeit with inferior mechanical properties [29,31]. Vacuum filtration provides greater control over thickness and is not also viable for large-scale production [3,29,31]. Additionally, investigations have examined the integration of diverse materials to improve the attributes of separators, including mechanical strength and ionic conductivity. It has been noted that the origin of cellulose significantly impacts its properties and performance, which varies according to the specific type of derivative (such as CNF, CNC, AC, CB, or RC) or the modifications applied. A comprehensive overview of the findings from the studies reviewed is presented in Table 3.

In the research conducted by Kumar et al. [32], cellulose derived from coffee waste was identified as a promising candidate, presenting a feasible substitute for commercial separators utilized in electric vehicles and smart devices. The extraction methodology comprised the collection, washing, drying, and grinding of the waste material, followed by an alkaline treatment and oxidation using the TEMPO reagent. Subsequent purification and dispersion processes yielded cellulose nanofibrils. Membranes were fabricated through a casting technique, employing a 2% concentration of cellulose nanofibrils in distilled water. These membranes were then applied onto a polytetrafluoroethylene (PTFE) substrate and subjected to drying in a vacuum oven at 45 °C for a duration of 720 min. The resulting membranes demonstrated a thickness ranging from approximately 10 to 25 µm, a porosity of 55%, exceptional thermal stability, and a notable enhancement in electrolyte absorption, reaching 266%. Furthermore, the biomass-derived separator exhibited improved ionic conductivity measured at 3.10–3 S.cm^−1^.

**Figure 3:**
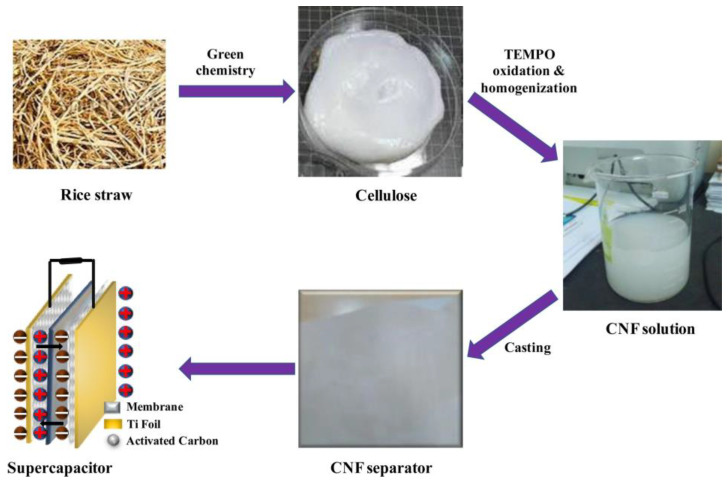
Schematic representation of the preparation of the supercapacitor. The membrane used as a separator is made by casting a mixture of cellulose nanofibrils and distilled water onto a PTFE plate The Figure was obtained by kind permission from [20].

**Figure 4:**
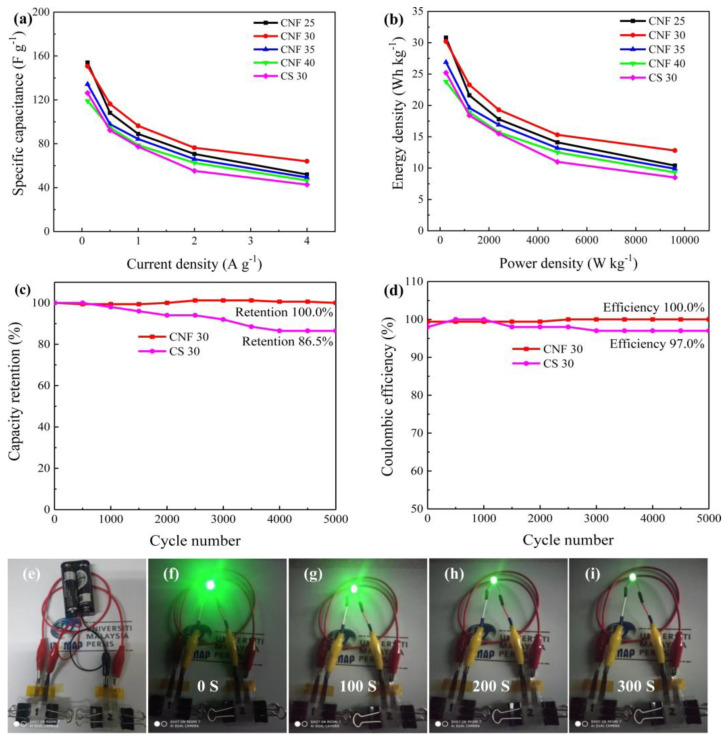
(**a**) Specific capacitance at different current densities from GCD profiles, (**b**) Ragone plot (power density vs. energy density) of different thickness CNF and CS 30, long–term cycling performances (**c**) capacity retention (**d**) coulombic efficiency of CNF 30 and CS 30 membranes, (**e**–**i**) photographic images of LEDs powered by the assembled CNF 30 device. The legend is the same as in the original study. The figure was obtained by kind permission from [20].

Islam et al. [20] employed a comparable methodology utilizing rice straw, as illustrated in Figure 3. Following the preparation of cellulose nanofibers, membranes were fabricated through the casting technique, utilizing CNF solutions with concentrations between 0.5% and 0.8%, which were dispersed in distilled water and shaped within PTFE plates measuring 12 × 12 cm. The resulting membranes had a thickness between 25 and 40 µm, with the 30 µm membrane being the most promising, showing porosity of 51%, specific capacitance of 150.7 F.g^−1^, energy density of 30.2 W.h.kg^−1^, and power of 240 W.kg^−1^. These values exceeded those of the 30 µm commercial separator (Titanium, The Woodlands, TX, USA) by 1.5 times. Moreover, the membrane exhibited excellent electrochemical stability, retaining 100% of its capacity after 5000 cycles, while the commercial separator retained only 86.5%. The performance of the supercapacitors is demonstrated in Figure 4.

**Figure 5:**
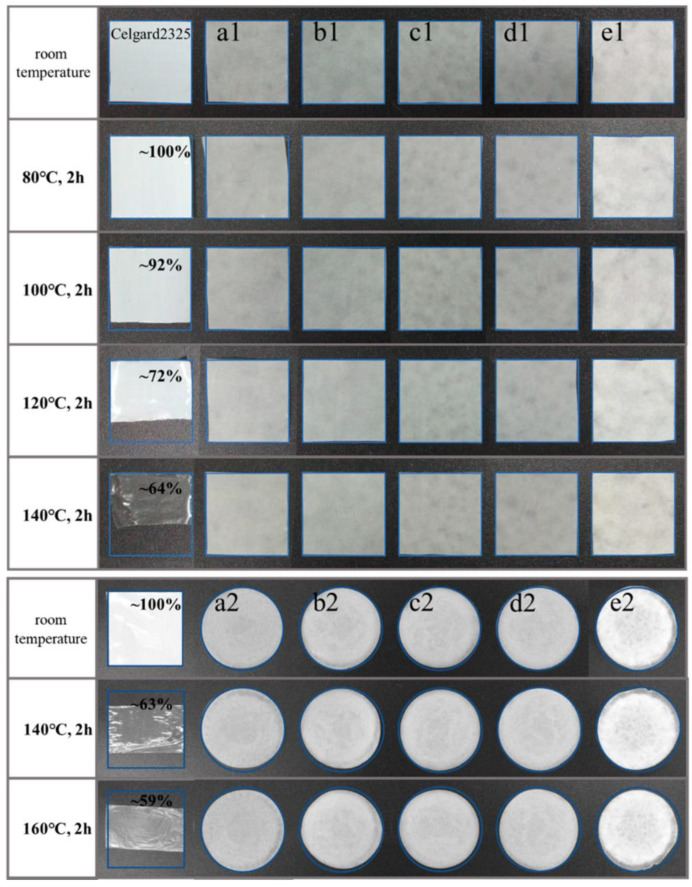
The thermal dimensional stability of Celgard 2325 and the cellulose membranes before and after the homogenization: (**a1**,**a2**) bamboo pulp; (**b1**,**b2**) hardwood pulp; (**c1**,**c2**) softwood pulp; (**d1**,**d2**) cotton pulp; (**e1**,**e2**) hemp pulp. The legend is the same as in the original study. The figure is reused under the Creative Commons Attribution (CC BY) license from [33].

Sheng, Wang, and Yang [33] developed membranes utilizing various cellulose sources, including bamboo, hardwood, softwood, cotton, and hemp, through a vacuum filtration method. Initially, each cellulose pulp was dispersed at a concentration of 10% (*w*/*w*) and processed using a PFI beater. Subsequently, the cellulose was suspended in a 1.0% (*w*/*w*) solution of distilled water, followed by ultra-fine friction milling, and then diluted to a concentration of 0.5% (*w*/*w*). This sequence of operations facilitated the production of nanocellulose. The resulting suspensions were subjected to vacuum filtration to form membranes with pore sizes of 0.45 µm, which were then dried in a vacuum oven, yielding membranes with thicknesses ranging from 21 to 23 µm, closely matching the commercial separator Celgard 2325 (24 µm). The contact angle measurements for the CNF membranes with distilled water were recorded at less than 45°. Furthermore, these membranes maintained their structural integrity at temperatures of 160 °C for a duration of 120 min. Notably, the nanocellulose membranes exhibited contact angles below 45°, with the hemp and hardwood pulp membranes demonstrating superior wettability at 18° and 21°, respectively. The softwood and cotton pulp membranes exhibited the highest tensile strength, surpassing 130 MPa. The authors concluded that these nanocellulose membranes are promising candidates for use as separators in lithium-ion batteries, owing to their remarkable wettability, thermal stability, and mechanical strength, although it is important to note that electrochemical testing was not performed. Figure 5 illustrates the thermal dimensional stability of the cellulose types examined.

Liu et al. [34] created hybrid separators that integrate hydroxyapatite (HAP) and cellulose nanocrystals (CNCs). The CNCs were derived from the oxidation of cellulose fibers using the TEMPO reagent, while hydroxyapatite was synthesized through a hydrothermal method utilizing calcium oleate as a precursor. Following the synthesis, the materials were combined, and membranes were produced via vacuum filtration. The resulting hybrid separators demonstrated remarkable thermal stability, capable of withstanding temperatures up to 250 °C, and exhibited flame-retardant characteristics. Additionally, their wettability with the electrolyte was notably superior, presenting a contact angle of 17.2°, in contrast to 45.5° for the conventional polypropylene separator. Furthermore, these separators outperformed in battery applications, with cells utilizing HAP/CNC achieving a capacity retention rate of 67.1% at 2C, surpassing the 57.8% retention rate of cells with polypropylene. Consequently, the authors successfully developed a hybrid separator utilizing sustainable raw materials and a solvent-free production process, positioning the HAP/CNC separator as a viable alternative for lithium-ion batteries.

Gonçalves et al. [35] fabricated cellulose nanocrystal films through the evaporation-induced self-assembly technique. This process involved the direct casting of cellulose nanocrystals, which were dispersed in water, into polystyrene Petri dishes. The resulting cellulose nanocrystal membranes exhibited a significant specific surface area and a porous three-dimensional structure, making them suitable for use as separators in battery applications. The membranes also displayed low contact angles with conventional electrolytes and ionic liquids, indicating that their 3D porous structure promotes wettability. Additionally, they exhibited ionic conductivity of 2.7 mS.cm^−1^, good electrochemical stability with specific capacity of 122 mAh.g^−1^ at a rate of C/2, and 85 mAh.g^−1^ at 2C.

**Figure 6:**
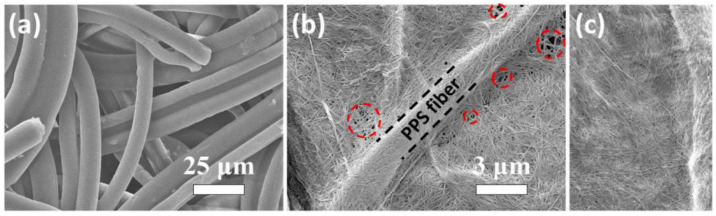
SEM images of PPS membrane: (**a**) 15% BC/PPS separator (**b**) and 20% BC/PPS separator; (**c**) the legend is the same as in the original study. The red circles represent some detected failures in the hybrid separator. The figure was obtained by kind permission from [36].

**Figure 7:**
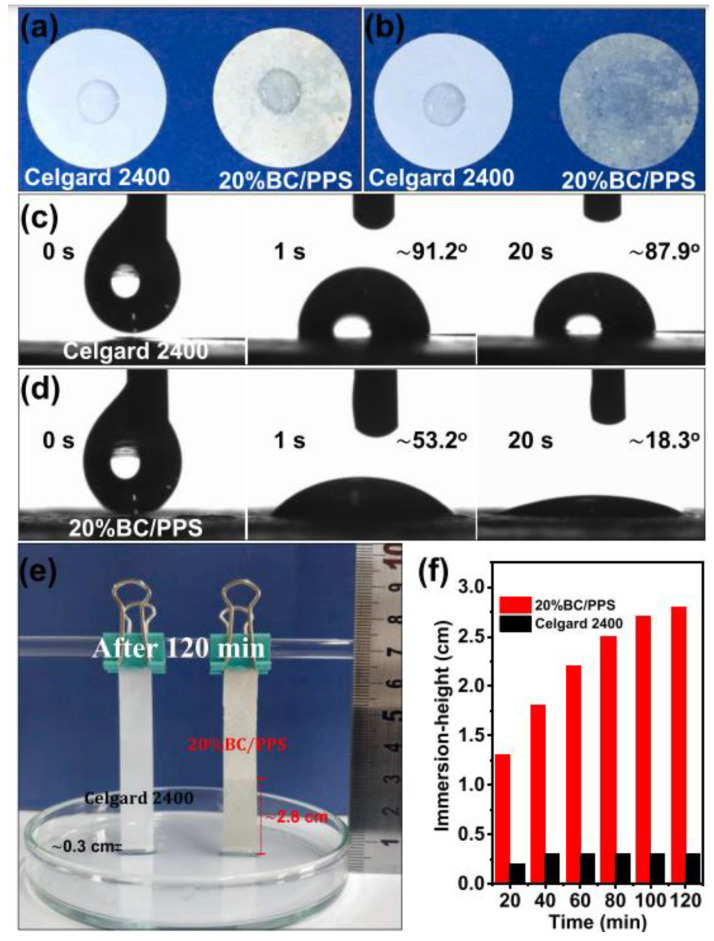
The wettability analyses of separators. Images of liquid electrolyte wetting behavior (**a**,**b**), contact angle variation photographs of electrolyte drop on different separators (**c**,**d**), the corresponding photo of electrolyte capillary absorption height after soaking for 120 min (**e**), and the record of height changed with time (**f**). The legend is the same as in the original study. The figure was obtained by kind permission from [36].

In the study conducted by Zhu et al. [36], a hybrid separator was created via vacuum filtration, incorporating heat-resistant polyphenylene sulfide (PPS) fibers alongside bacterial cellulose nanofibers (CB/PPS) (see Figure 6). The findings indicated remarkable thermal stability, which can be ascribed to the elevated melting point of the polymer and the significant crystallinity present in the cellulose chains of the bacterial cellulose. Furthermore, the hybrid separator CB/PPS with 20% CB exhibited good wettability with electrolyte and ionic conductivity (1.55 mS.cm^−1^) (Figure 7). The battery cells using the hybrid separator showed a higher rate capacity and a more stable cycling performance compared to those using commercial separators.

Huang et al. [37] conducted a study on the fabrication of oxidized bacterial cellulose nanofiber membranes intended for use as separators in lithium-ion batteries. These membranes were created from an aqueous dispersion of bacterial cellulose nanofibers through the vacuum filtration technique. The resulting membranes demonstrated sufficient porosity and a strong affinity for the liquid electrolyte and lithium electrode, which contributed to enhanced electrolyte absorption and reduced interfacial resistance. Notably, the membrane derived from a 1.0% bacterial cellulose dispersion exhibited remarkable properties, including an electrolyte absorption rate of 339% and an ionic conductivity of 13.45 mS.cm^−1^, alongside impressive cycling stability, maintaining a capacity retention of 94% after 100 cycles at a rate of 0.2C.

Chen et al. [38] developed hybrid separators composed of polyacrylonitrile (PAN) combined with cellulose acetate and hydroxyapatite nanoparticles. The authors first created a pure PAN membrane through the electrospinning process, followed by the application of cellulose acetate and hydroxyapatite coatings using a coating technique. The resulting hybrid separator, designated as PAN/AC/HAP with 1.0% TEMPO, demonstrated remarkable thermal resistance and significant dimensional stability. Due to the hydrophilicity of AC and the large specific surface area of HAP, the wettability of the electrolyte was enhanced, resulting in increased ionic conductivity (3.02 mS.cm^−1^). In the charge and discharge performance tests, the hybrid separator achieved a high discharge capacity of 161 m.A.h.g^−1^, maintaining excellent cycling stability.

**Figure 8:**
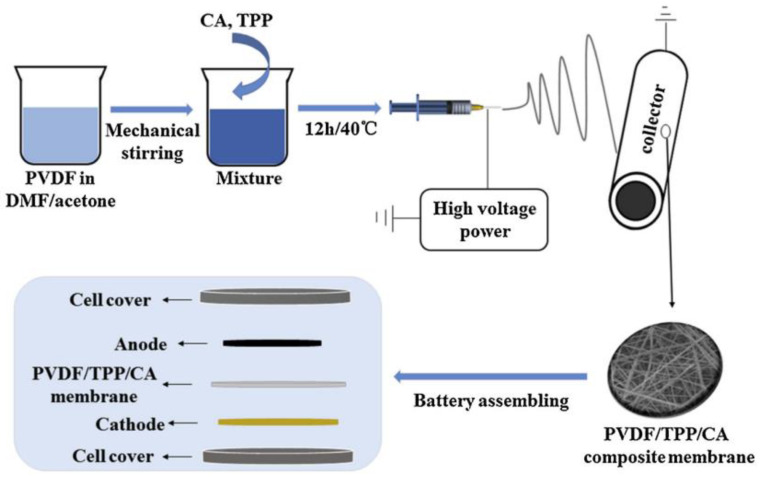
Schematic representation of the supercapacitor production. The figure was obtained by kind permission from [39].

In the research conducted by Chen et al. [39], a hybrid separator was fabricated utilizing a membrane composed of polyvinylidene fluoride (PVDF), triphenyl phosphate (TPP), and cellulose acetate (CA) through the process of electrospinning (see Figure 8). The resulting separator displayed notable characteristics, including a porosity of 90%, enhanced thermal stability, improved electrolyte wettability (14.6°), significant electrolyte absorption (301%), and superior flame resistance. Furthermore, batteries constructed with the PVDF/TPP/CA membrane exhibited remarkable electrochemical performance and cycling stability, achieving a capacity retention of 86.9% after 100 cycles. These superior attributes are primarily attributed to the membrane’s porous architecture and the synergistic interactions between CA and TPP.

Wang et al. [8] introduced an innovative separator derived from cotton pulp through the non-solvent phase inversion process (NIPS). The initial step involved dissolving cotton pulp in a solution comprising 7 wt% sodium hydroxide, 12 wt% urea, and 81 wt% water, which was pre-cooled to −12 °C and subsequently subjected to centrifugation. This process yielded a transparent solution of regenerated cotton cellulose at a concentration of 2.5% (*w*/*w*). It is crucial to emphasize that the cellulose solution utilized in this study is not in a nano-sized form; rather, it is a regenerated cellulose solution that is conducive to film formation. The separator membrane was fabricated by applying the cellulose solution onto a glass plate with a doctor blade, followed by immersion in an ethanol coagulation bath for 15 min and a thorough wash in distilled water for 48 h to eliminate any residual ethanol. The resulting separators demonstrated remarkable thermal stability, enduring temperatures up to 180 °C, alongside a substantial electrolyte absorption capacity of 436%. Additionally, they exhibited ionic conductivities ranging from 0.9 to 1.25 mS.cm^−1,^ exceptional electrochemical stability, and enhanced cycling performance when compared to the commercial separator Celgard 2325, achieving discharge capacities of 110–117 m.A.h.g^−1^ and a retention rate of 76–81% after 100 cycles, in contrast to 100 m.A.h.g^−1^ and 72% for the commercial counterpart.

In the research conducted by Guo et al. [40], a thermally stable and eco-friendly separator was developed, utilizing a combination of regenerated cellulose and polyvinyl alcohol (PVA). The cellulose was derived from cotton pulp through a process involving urea, sodium hydroxide, and water, mirroring the methodology outlined in reference [7]. The membranes were fabricated using the phase inversion technique, applied with a knife to achieve a uniform thickness of 0.4 mm, and subsequently immersed in PVA solutions. The resulting separators demonstrated significant porosity, favorable wettability, a tensile strength of 53.7 MPa, and minimal thermal shrinkage at 200 °C. With an ionic conductivity measured at 1.34 mS.cm^−1^ and outstanding interfacial compatibility, the authors concluded that the separators made from regenerated cellulose and PVA present a viable alternative to commercial options, characterized by enhanced safety, cost-effectiveness, and biocompatibility.

Based on the analysis of the 11 articles reviewed, it is clear that each type of cellulose derivative has a significant effect on the performance of battery separators. In addition, the processing method plays a crucial role in determining the electrochemical performance. In this review, techniques such as electrospinning and vacuum filtration were shown to improve electrochemical performance by increasing the uniformity and porosity of the separators, which in turn increases ion transport and electrolyte absorption [12]. Among the cellulose derivatives, cellulose acetate and bacterial cellulose have received considerable attention due to their favorable electrochemical performance and are the most studied by the scientific community. However, as mentioned above, these derivatives often require chemical solvents or involve expensive and time-consuming production processes, such as bacterial cellulose synthesis, which pose challenges for large-scale industrial applications.

In contrast, nanocellulose is a promising alternative due to its ease of processing and sustainable properties. Recent studies, such as the work of Kumar et al. [20], have shown that even materials derived from agricultural waste, such as rice straw, combined with simple techniques such as casting with distilled water, can achieve exceptional electrochemical performance. These advances highlight the potential of nanocellulose separators to achieve a balance between high performance and environmental sustainability, offering a viable solution for the future of energy storage technologies.

## 4. Environmental Impact and Sustainability of Cellulose-Derived Battery Separators

The life cycle of separators can be categorized into three primary phases: production, utilization, and disposal/recycling. The production phase includes the extraction of raw materials, manufacturing processes, and transportation logistics. The utilization phase pertains to the operational function of the separator within a battery system. Lastly, the disposal and/or recycling phase addresses the management of the separator once it has reached the end of its operational lifespan. Life cycle assessment (LCA) serves as a methodological framework for analyzing the environmental impacts associated with a product throughout its entire life cycle [41,42]. In the context of separators, key performance indicators evaluated include energy efficiency, which is affected by ionic conductivity; durability, which is quantified by the number of charge and discharge cycles the separator can endure before degradation occurs; and the carbon footprint of each separator, which is assessed based on CO_2_ emissions generated during the aforementioned phases of production, utilization, and disposal/recycling [41,42]. The increasing demand for lithium-ion batteries (LIBs), coupled with various life cycle challenges, including battery degradation processes, energy usage, greenhouse gas (GHG) emissions, and the depletion of raw materials, underscores the necessity for comprehensive sustainability evaluations to assess the environmental impacts associated with these batteries [43].

The quest for enhanced sustainability in technology has catalyzed the investigation and innovation of alternative materials for critical components of batteries, particularly separators. Historically, separators made from polypropylene and polyethylene have been prevalent, primarily due to their affordability and the feasibility of mass production. Nevertheless, these materials originate from non-renewable resources, rendering them non-biodegradable and resulting in prolonged decomposition periods [44,45,46,47,48]. In contrast, cellulose separators offer an environmentally friendly profile, as they are derived from renewable sources and are biodegradable, thereby reducing environmental impact at the end of their life cycle [49,50]. Studies indicate that replacing synthetic separators with cellulose can reduce the carbon footprint by up to one-third [51]. This is due to both the use of less energy-intensive processes and the reduction of non-recyclable plastic waste.

Although defibrillation, regeneration, and oxidation processes consume energy and can generate waste, these renewable and biodegradable materials significantly reduce the environmental impact at the end of their life cycle [52]. These separators have lower carbon emissions and lighter, more efficient designs, reducing energy consumption and supporting a circular economy [39,52,53,54].

In addition to environmental benefits, cellulose separators display superior thermal stability, as they are highly stable at elevated temperatures without undergoing shrinkage or melting, which reduces the risk of thermal failures and enhances battery safety [49,50]. Cellulose exhibits favorable wettability with electrolytes, which enhances its electrochemical characteristics and, as a result, boosts energy efficiency. It is frequently employed in hybrid separators, where it is integrated with various materials to improve essential properties, including mechanical strength, ionic conductivity, and thermal stability [50]. Cellulose separators, while still in the preliminary phases of investigation and necessitating further validation for large-scale commercial application, offer a promising and sustainable alternative for the energy storage sector. They align with the growing technological and environmental requirements that are becoming increasingly rigorous.

Recent studies on cellulose-derived separators focus on their electrochemical efficiency and durability during use. While the use of cellulose in energy storage devices has gained attention, there is a notable lack of research on the life cycle assessment of these materials, which remains a gap to be explored in future studies. Although LCA is a valuable tool for identifying critical environmental impact points and proposing strategies to mitigate these effects, the current body of work on LCA in the context of lithium-ion batteries remains limited [41,42,43]. Most studies focus on the production phase of LIBs, with relatively little attention given to the use and disposal phases [43].

Zhang et al. [51] developed a nonwoven separator made from cellulose diacetate (CDA) through electrospinning. To enhance the mechanical properties, polyethylene oxide (PEO) was incorporated, and boehmite (BM) was added at various mass concentrations (0 wt%, 0.5 wt%, 1.0 wt%, and 1.5wt%). Among the formulations tested, the nonwoven composite M-CDA/PEO/BM-1 (with 1.0 wt% BM) stood out as an eco-friendly, low-cost, recyclable, and highly promising solution for lithium-ion battery separators. The use of modified CDA as the separator base material improved thermal and electrochemical performance, overcoming typical issues found in polyolefin separators. Compared to the commercial polypropylene (PP-Celgard 2400) separator, M-CDA/PEO/BM-1 exhibited electrolyte absorption of 230.2% and ionic conductivity of 2.83 mS.cm^−1^, while the commercial separator showed lower values with 150.2% and 0.934 mS.cm^−1^, respectively. Regarding thermal stability, the PP separator showed 90% shrinkage at 200 °C, while the hybrid M-CDA/PEO/BM-1 separator showed no dimensional changes at 200 °C, demonstrating superior heat resistance. The electrochemical performance was also analyzed. After 100 cycles at 0.5C, the discharge capacity of M-CDA/PEO/BM-1 was 170.3 mAh.g^−1^, surpassing the 123.2 mAh.g^−1^ of the PP separator. The results indicate that the cellulose separator significantly enhances the battery’s cyclic performance due to its greater electrochemical stability. The findings highlight that cellulose-derived separators, such as the innovative M-CDA/PEO/BM-1 composite, offer advantages over polypropylene separators throughout their life cycle. In terms of LCA, it can be concluded that during the production stage, the lower carbon emissions associated with the use of renewable raw materials and less energy-intensive processes contribute to a reduced environmental footprint. During use, the superior performance is demonstrated by higher energy efficiency, driven by better ionic conductivity and thermal stability, as well as greater durability, measured by stability over charge and discharge cycles. In summary, M-CDA/PEO/BM-1 not only improves lithium-ion battery performance but also represents an environmentally responsible solution, integrating operational efficiency with reduced environmental impact throughout all stages of the life cycle.

The study conducted by Ratri et al. [55] investigated the effects of using succinonitrile and citric acid as plasticizers in the production of cellulose-derived polymeric membranes. The membranes were produced using the casting method, with dimethyl sulfoxide (DMSO) as a solvent, presenting a green approach in the manufacturing process. The results indicated that the plasticized cellulose membranes exhibited enhanced dimensional stability and thermal resistance, key characteristics for improving the safety of lithium-ion batteries. In contrast, the commercial separator deformed at 120 °C, while the cellulose membranes maintained their structural integrity. The maximum ionic conductivity recorded was 2.73 × 10^−5^ S.cm^−1^, achieved with 6% succinonitrile as a plasticizer. This value represents a substantial improvement over the commercial separator, Celgard 2325 (trilayer PP/PE/PP membrane), which showed a conductivity of 2.14×10^−7^ S.cm^−1^. The results demonstrated better electrochemical efficiency and thermal resistance for the cellulose separator compared to the commercial separator. Furthermore, the use of green solvents in the manufacturing process emphasizes the commitment to reducing environmental impact, solidifying this approach as a viable and sustainable alternative for the development of high-performance separators.

Ren et al. [56] developed a cellulose-derived separator with flame-retardant properties, named CBS@H-AP, to enhance the safety and cycle stability of lithium-ion batteries. CBS@H-AP was synthesized through a crosslinking reaction between H-AP (4-aminopyridine and hexachlorocyclotriphosphazene) and the cellulose surface, resulting in a structure with excellent wettability, high porosity, good electrolyte absorption, as well as remarkable flame-retardant and thermal stability properties. In terms of performance, the CBS@H-AP separator demonstrated low flammability and high dimensional thermal stability, showing no significant shrinkage at 240 °C for 5 min. The use of the CBS@H-AP separator resulted in excellent cycle stability, with a capacity retention of 97.2% and a coulombic efficiency of 99.2% at 0.5C after 100 cycles. This study opens new possibilities for the application of cellulose-derived separators, improving not only the safety but also the cycle stability of lithium-ion batteries.

Chen et al. [57] reported the creation of a hybrid cellulose membrane that is coated with PVDF-HFP (polyvinylidene fluoride-co-hexafluoropropylene) and LATP (lithium aluminum titanium phosphate) utilizing the doctor-blading coating technique. The membrane was formulated with a LATP:PVDF-HFP weight ratio of 9:1. This methodology sought to integrate the thermal and electrochemical characteristics of the coatings with the sustainability and efficacy of cellulose. The findings revealed that the hybrid membranes exhibited remarkable thermal stability, in contrast to the commercial polypropylene (PP) separator, which experienced shrinkage at 150 °C for 30 min and completely melted at 200 °C. In comparison, both the pure cellulose and hybrid cellulose membranes maintained their dimensions without any shrinkage or deformation. Regarding ionic conductivity, the commercial PP membrane demonstrated a value of 0.77 mS.cm^−1^, while the cellulose membrane achieved 1.11 mS.cm^−1^, and the hybrid membrane reached 2.13 mS.cm^−1^. In electrochemical performance evaluations conducted at 0.2C over 200 cycles, the battery utilizing the PP separator recorded an initial discharge capacity of 144.3 mAh.g^−1^, whereas the battery with the cellulose membrane achieved 157.8 mAh.g^−1^, and the hybrid membrane exhibited a capacity of 142.9 mAh.g^−1^. After 200 cycles, the hybrid membrane retained 86.7% of its initial capacity, underscoring its durability. These results suggest that both pure and hybrid cellulose separators surpass commercial PP separators in terms of thermal stability, ionic conductivity, and cycling capacity.

Throughout their life cycle, cellulose separators help reduce the carbon footprint. From production, where renewable raw materials reduce emissions, to their improved performance in energy storage devices resulting in longer lasting batteries, to their environmentally friendly disposal or recycling options, cellulose separators meet the growing demand for green technologies. Integrating LCA into future research will be crucial to further optimizing these materials and ensuring that their environmental benefits are fully realized.

## 5. Conclusions

With the increasing environmental concerns and the search for more sustainable technologies, cellulose, a renewable and biodegradable resource, emerges as a promising alternative for battery separators. In addition to reducing the environmental impact, cellulose improves the thermal properties, safety, efficiency, and performance of batteries. Despite the challenges faced in the production and application of cellulose derivatives, research indicates that with the development of more efficient processes and the incorporation of additives, it is possible to improve the commercial viability of these materials. The environmental analysis presented in this article highlights the reduction in carbon footprint associated with cellulose-derived separators, emphasizing their contribution to more sustainable energy storage solutions. Thus, cellulose separators have the potential to transform the energy storage sector, promoting a circular economy and meeting current demands for sustainable technological solutions.

## 6. Challenges and Perspectives

Cellulose-derived battery separators represent a promising alternative to commercial separators, providing a more sustainable and efficient technology for the energy storage sector [12]. However, each form of cellulose has its own characteristics that influence its applications.

CNFs, for example, tend to agglomerate, which makes it difficult to create controlled porous structures, rendering the separators more fragile [10,11,12,21]. On the other hand, CNCs face the challenge of achieving uniform dispersion, which also compromises the mechanical strength of the separators [22]. Other cellulose derivatives, such as cellulose acetate, have limited chemical stability and may degrade over time in the battery, depending on the electrolyte used [49]. Bacterial cellulose requires controlled production conditions and long cultivation periods, making its industrial scalability impractical [25,26,58,59]. Finally, regenerated cellulose exhibits high hygroscopicity, easily absorbing water, which can compromise ionic conductivity and increase the risk of short circuits in humid environments [8,60]. A solution to these problems is the incorporation of additives or the combination of cellulose in hybrid separators, which can enhance the properties of the components, particularly regarding mechanical strength, porosity, chemical stability, and flame-retardant properties [23,61,62].

Despite the promising work presented in this review, the use of cellulose derivatives in industrial applications still faces challenges, primarily in developing scalable and economically viable processes [63]. The main methods used to prepare high-performance cellulose-based separators include electrospinning, vacuum filtration, and phase inversion. Electrospinning is a time-consuming and costly process, limiting its production efficiency [64]. Filtration, while simple and easy to apply, is not suitable for large-scale production [65]. Phase inversion involves many parameters and experimental conditions, making the process challenging [66].

In this scenario, there is an opportunity for innovations in research aimed at optimizing large-scale production technologies for cellulose components for electronic devices. It is essential to develop simple, environmentally friendly and cost-competitive solutions. Emerging trends in the development of cellulose-based separators focus on sustainable processing and innovative material combinations. The use of green solvents [55], water-based processing methods [20,32,33,35], and recycling of agro-industrial byproducts [20,32] is gaining traction as a means to make cellulose manufacturing more environmentally friendly. Furthermore, research into flame-retardant innovations [34,39,56] is contributing to the safety and performance enhancement of these separators, with the goal of meeting the growing demand for energy storage systems that are both safe and efficient. In addition, the use of 3D printing technology to fabricate separators offers a promising approach. This method allows the production of separators with flexibility that can accommodate large deformations, improving both the mechanical flexibility and electrochemical stability of energy storage devices [67].

In summary, cellulose-derived battery separators have the potential to revolutionize the energy storage sector by replacing synthetic materials with renewable and environmentally safe alternatives. The use of cellulose derivatives addresses the growing demand for more sustainable technologies, shaping the future of next-generation energy storage systems.

## Figures and Tables

**Table 1:** Main requirements for battery separators (see ref. [3]).

Parameter	Objective
Pore size	<1.0 µm
Porosity	40.0–60.0%
Thickness	20.0–25.0 µm
Tensile strength	98.06 (>1000 kg.cm^−1^) MPa
Thermal shrinkage	<5.0% at 90 °C for 60 min
High-temperature melt integrity	>150 °C
Ionic conductivity	10^−3^–10^−1^ S.cm^−1^

**Table 2:** Main advantages and disadvantages of cellulose derivatives as battery separators.

Type of Cellulose	Production Method	Advantages	Disadvantages
CNF	Mechanical defibrillation	Enhances mechanical strength, flexibility, thermal stability, ionic conductivity, and electrolyte retention	Tendency to aggregate, structural fragility
CNC	Acid hydrolysis and oxidation (e.g., TEMPO reagent)	Provides matrix reinforcement, better control of porosity, and improved ionic conductivity	High cost
BC	Synthesized by bacteria (e.g., *Gluconacetobacter xylinus*)	Excellent thermal stability, long cycle life, and potential for energy efficiency improvement with conductive additives	Slow production process, high cost
CA	Esterification of cellulose with acetic acid and anhydride	Flexibility, good electrical insulation, and can be modified to improve electrochemical properties	Limited chemical stability
RC	Chemical coagulation and regeneration processes	Flexibility, porosity, and enhances battery performance and lifespan	High hygroscopicity

**Table 3:** Different cellulose-derived separators: findings from the literature.

Separator	Type of Cellulose	Methodology	Thickness (µm)	Porosity (%)	Ionic Conductivity (mS.cm^−1^)	Electrochemical Perfomance	Battery (Cathode/Anode)	Reference
**CNF**	Coffee waste	Casting	25	55	3.00	Specific capacitance retention of 47.1%	Zn/SS	[32]
**CNF**	Rice straw	Casting	30	51	3.40	100% after 5000 cycles at 0.5C	Activated carbon	[20]
**CNF**	Bamboo pulp, hardwood pulp, softwood pulp, cotton pulp, and hemp pulp	Vacuum filtration	20–30	-	-	-	-	[33]
**HAP/CNC**	CNC	Vacuum filtration	28	76	0.81	67.1% after 100 cycles at 2C	LiFePO_4_/Li	[34]
**CNC**	CNC	Casting	150	75.3	2.7	91 mAhg^−1^ after 10 cycles at C/8	LiFePO_4_/Li	[35]
**BC/PPS**	BC	Vacuum filtration	-	62.7	1.55	91.3% after 100 cycles at 0.5C	LiFePO_4_/Li	[36]
**TOBC**	BC	Vacuum filtration	29	88.3	13.45	94% after 100 cycles at 0.2C	LiFePO_4_/Li	[37]
**PAN/CA/HAP**	AC	Eletrospinning	46	61	3.02	157.6 mAhg^−1^ after 50 cycles at 0.5C	LiFePO_4_/Li	[38]
**PVDF/CA/TPP**	AC	Eletrospinning	58	90	4.4	86.9% after 100 cycles at 0.2C	LiFePO_4_/Li	[39]
**RCS**	Cotton pulp	Phase inversion	19.74	61	1.25	72% after 100 cycles at 0.2C	LiFePO_4_/Li	[8]
**CSA**	Cotton pulp	Phase inversion	109	58.43	1.34	78.7% after 80 cycles at 0.5C	LiFe_0.2_Mn_0.8_PO_4_/Li	[40]

CNF—cellulose nanofibrils, HAP—hydroxyapitite, CNC—cellulose nanocrystals, BC—bacterial cellulose, PPS—polyphenylene sulfide, TOBC—TEMPO-mediated oxidized cellulose nanofibers, PAN—polyacrylonitrile, CA—cellulose acetate, PVDF—polyvinylidene fluoride, TPP—tethered cellulose, RCS—regenerated cellulose scaffolds, CSA—polyvinyl alcohol modified cellulose/styrene-co-acrylate.

## Data Availability

The data are contained within the article.
